# Cascading lake drainage on the Greenland Ice Sheet triggered by tensile shock and fracture

**DOI:** 10.1038/s41467-018-03420-8

**Published:** 2018-03-14

**Authors:** Poul Christoffersen, Marion Bougamont, Alun Hubbard, Samuel H. Doyle, Shane Grigsby, Rickard Pettersson

**Affiliations:** 1 Scott Polar Research Institute, University of Cambridge, Cambridge, CB2 1ER UK; 2 Centre for Arctic Gas Hydrate, Environment and Climate, Department of Geology, The Arctic University of Norway, N-9037 Tromsø, Norway; 3 Centre for Glaciology, Department of Geography and Earth Sciences, Aberystwyth University, Aberystwyth, SY23 3DB UK; 4 Cooperative Institute for Research in Environmental Sciences, University of Colorado at Boulder, Boulder, CO 80309 USA; 5 Department of Earth Sciences, Geocentrum, Villavägen 16, 752 36 Uppsala, Sweden

## Abstract

Supraglacial lakes on the Greenland Ice Sheet are expanding inland, but the impact on ice flow is equivocal because interior surface conditions may preclude the transfer of surface water to the bed. Here we use a well-constrained 3D model to demonstrate that supraglacial lakes in Greenland drain when tensile-stress perturbations propagate fractures in areas where fractures are normally absent or closed. These melt-induced perturbations escalate when lakes as far as 80 km apart form expansive networks and drain in rapid succession. The result is a tensile shock that establishes new surface-to-bed hydraulic pathways in areas where crevasses transiently open. We show evidence for open crevasses 135 km inland from the ice margin, which is much farther inland than previously considered possible. We hypothesise that inland expansion of lakes will deliver water and heat to isolated regions of the ice sheet’s interior where the impact on ice flow is potentially large.

## Abstract (web summary)

Lakes on the Greenland Ice Sheet transfer water to the bed when they drain, but the impact is unknown. Here, the authors use a 3D model to show that lakes drain when fractures form, causing a chain reaction in which cascading lake drainages extend inland and deliver water to previously isolated regions of the bed.

## Introduction

Each summer thousands of surface melt lakes form across the Greenland Ice Sheet and those that drain abruptly^1–3^ cause short-lived^4–7^, yet pronounced accelerations in ice flow^8,9^ due to loss of basal traction^10,11^. These supraglacial lakes (SGLs) typically start to form in late May and they grow in number and extend as surface melting progresses to higher elevations during the melt season^1^. SGLs have become larger and more numerous since 2000^1,3^, while also expanding inland to elevations as high as 2000 m above sea level and 130 km inland from the ice margin^12^. The ability of these new inland lakes to enhance ice flow through rapid drainage is, however, contested^13^. Several studies suggest that lakes forming at high elevations tend to be larger and less likely to drain rapidly compared to lakes at lower elevations^1,2,14^ where extensional flow is capable of initiating hydro-fractures beneath lakes^15^. Hence, Poinar et al.^15^ argue that meltwater produced above 1600 m elevation predominantly drains on the surface and that the impact of inland SGL expansion therefore may be limited in terms of ice dynamical feedbacks. Yet, Doyle et al.^16^ argue that draining lakes may accelerate flow at 1840 m elevation and as far as 140 km inland from the margin. They also report year-on-year increases in ice flow corresponding to the expanding extent of SGLs, a response that differs fundamentally from the decadal slowdown observed closer to the margin^17,18^. With SGLs predicted to expand 200 km inland from the margin over the next 50 years^19^, it is critical to understand the lake drainage mechanism and its role in delivering surface water to the interior bed, where basal drainage is thought to be predominantly inefficient^20–22^ and the impacts therefore potentially sustained^16^.

Here we apply a well-constrained, three-dimensional (3D) ice-flow model of the Kangerlussuaq sector of the Greenland Ice Sheet to test the hypothesis that SGL drainage is dynamically triggered by the perturbation induced on the force balance of the ice sheet when surface meltwater is routed along the bed in summer. We show that distinct events, with up to 124 lakes draining over the course of a few days, occur when basal lubrication along subglacial drainage paths transiently induces high-magnitude tensile stresses near the surface. This ephemeral and previously overlooked alteration of the ice sheet’s force balance escalates into a tensile shock when many lakes drain collectively in a chain reaction. We use cascading lake drainage to describe the latter and show that most lakes drain in this dynamic manner.

## Results

### Ice sheet model

**Fig. 1:**
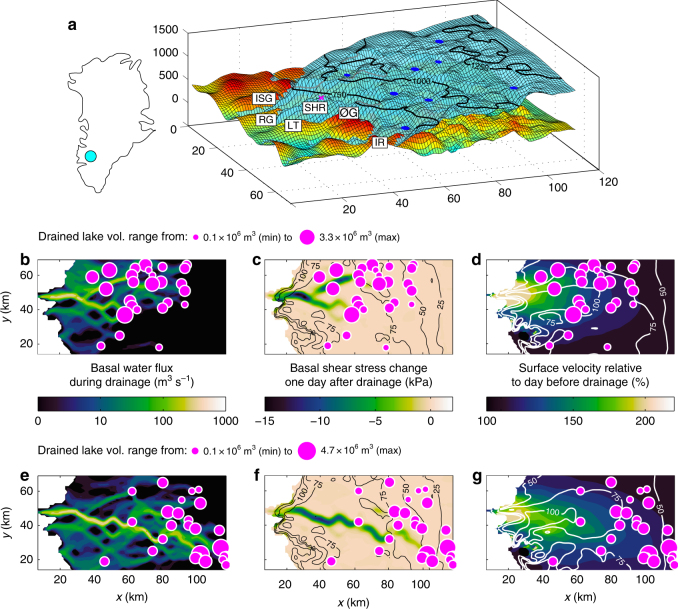
Cascading supraglacial lake drainage events. **a** Numerical model of the Kangerlussuaq sector of the Greenland Ice Sheet, including Isunnguata Sermia (ISG), Russell Glacier (RG), Leverett Glacier (LT), Ørkendalen Glacier (ØG), Isorlersuup Glacier (IR) and site SHR on the K-transect. **b** Flux of water in the basal drainage network on 21 June when 26 lakes drained rapidly. Solid magenta dots denote location and volume of lakes, which lost between 0.1 and 3.3 million cubic metres of water when they drained. **c** Change in basal shear stress one day after drainage when the ice sheet has responded to the lubrication of flow by surface water injected at the bed. Black contours show absolute values of basal shear stress prior to drainage. **d** Surface velocity relative to day before drainage. White contours show absolute values of surface velocity prior to drainage. **e**–**g** same as **b**–**d** but for cascading lake drainage on 18 July

**Fig. 2:**
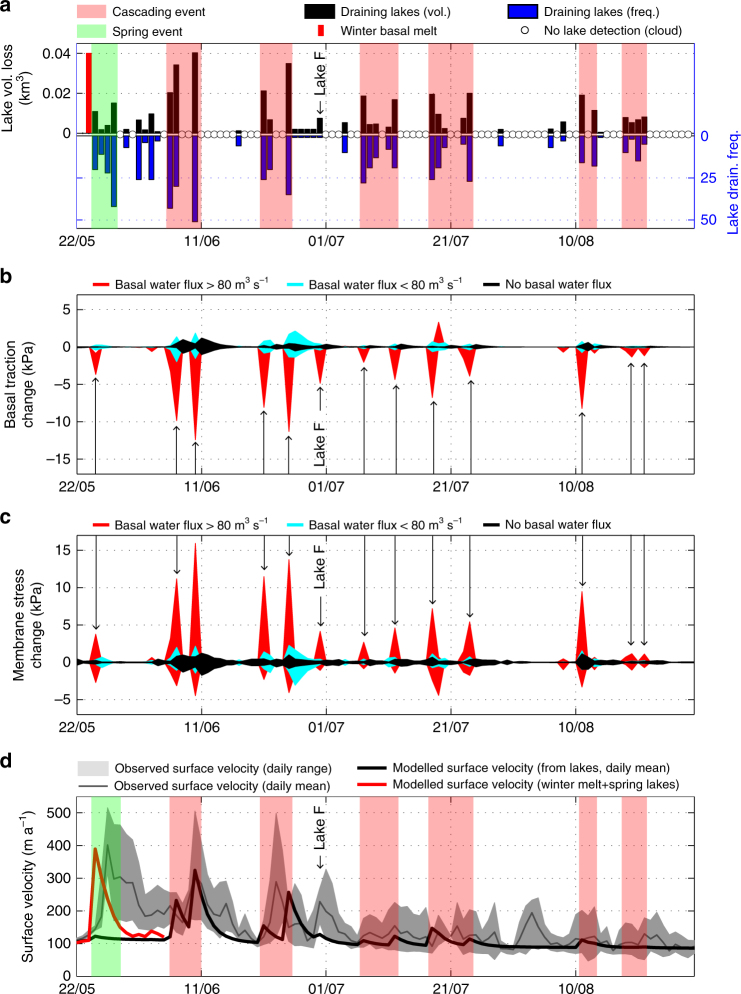
Lake drainage and activation of the membrane stress. **a** Supraglacial lake drainage record used to force numerical model (Data from Fitzpatrick et al.^1^). Black (blue) bars show volume (frequency) of draining lakes. Red bar shows modelled quantity of basal meltwater produced in winter and assumed to be released when the first lakes drain. Green shaded area shows a spring speed-up event. Shaded red areas outline cascading events which occur when lakes drain in rapid succession, causing ice flow to transiently accelerate. **b** Change in basal traction averaged over regions of the bed where the basal water flux in grid cells of model is high (>80 m^3^ s^−1^, red), moderate (<80 m^3^ s^−1^, teal) and nil (black). The change is averaged across sections of the bed and is relative to day before. Arrows mark major perturbations at high-flux drainage paths and Lake F refers to the drainage event reported by Doyle et al. (ref. ^6^). **c** Same as **b** but showing change in the membrane stress within the ice sheet. Arrows mark the transient activation of the membrane stress along high-flux drainage paths along the bed. Note how changes in membrane stress correspond to changes in basal traction. **d** Mean daily surface velocity recorded at site SHR (67.099°N, 49.936°W) with GPS (grey solid line with shading showing range of daily variation) and modelled daily surface velocity at the same location (black solid line) when ice sheet model is forced by lake volumes shown in **a**. Model explains 83% of summer observations spanning June, July and August. Red solid line shows spring speed-up event simulated in a separate experiment in which 0.04 km^3^ of basally produced meltwater (red bar in **a**) is stored subglacially in winter, when the terminus freezes, and released when the first lakes begin to drain in May (see Methods)

We apply the higher order Community Ice Sheet Model (CISM) to a 9000 km^2^ domain that extends 110 km inland from ice sheet margin near Kangerlussuaq (67.10°N, 49.90°W) and includes five outlet glaciers (Isunnguata Sermia, Russell, Leverett, Ørkendalen and Isorlersuup glaciers in West Greenland) (Fig. 1a). Model spin-up conditions were specified by inversion of observed winter 2009–2010 surface velocities^23^, which yield a robust fit (*r*^2^ = 0.99, *p* < 0.01) between observation and the initialised model (Supplementary Fig. 1). We then forced the model with the record of 156 lakes, which transferred 0.43 km^3^ of water to bed of the ice sheet during 663 individually observed events in 2010 (ref. ^1^; Fig. 2a). The water from draining SGLs was injected at the bed beneath each lake and then routed subglacially in a basal hydrological system. The latter was coupled to a 5-m-thick layer of soft basal till, which is a glacially produced sediment observed beneath the ice sheet in this region^24–26^ including lake sites^26^. In this model set-up, basal traction was specified by the till layer’s shear strength, which evolved according to vertical water flow within it as well as through exchange of water with a basal hydrological system transporting water according to the gradients of the hydro-potential surface (Methods). Although the total runoff produced by surface melting in 2010 was 6.58 km^3^ (ref. ^27^) and therefore considerably higher than the amount of water stored in SGLs, we focus on the latter because previous work found lake drainages to induce short-lived but sustained episodes of ice flow acceleration, consistent with the observed seasonal variation of ice flow in this^11^ and other sectors^8^ of the Greenland Ice Sheet. Although a larger quantity of surface meltwater is transferred to the bed through moulins forming where lakes have drained and where supraglacial streams intersect open crevasses, we exclude this water supply because its variability is insufficient to drive a sustained response in the ice flow of our model^11^.

### Cascading lake drainage

Modelled ice flow varies spatially and temporally in a 6-month forward simulation, which starts on 15 May. Although a significant number of lakes drain during 25–28 May (Fig. 2a), our model does not initially reproduce the first speed-up event observed during that period (Fig. 2d). To generate this 'spring event', we included meltwater produced by friction at the base of the ice sheet during winter and released it when the first lakes drain on 25 May (Fig. 2d) (see ref. ^11^ and Methods). From 1 June and onwards, the lake drainage record produces distinct intra-seasonal variations in our model, with flow rapidly accelerating by up to 400% compared to winter. The sustained, but short-lived perturbations generated by draining lakes are in good general agreement with daily ice-flow variations recorded by a GPS receiver installed at site SHR on Russell Glacier (Fig. 2d). The model outputs are also consistent with ice flow observed more broadly in satellite remote imagery from 19 June, 11 July, 22 July and 11 November (Supplementary Fig. 2). In a previous study we used this validated model set-up to show how basal properties change when SGLs drain and flood the bed^11^; here, we examine how the force balance of the ice sheet is altered when basal traction is temporarily lost. To understand the dynamic response to multiple lakes draining on the same day, we use an observationally well-constrained event on 21 June, when 26 lakes drained and transferred 21 × 10^6^ m^3^ of surface meltwater to the bed. After that event, water fluxes in the subglacial drainage system of our model were >80 m^3^ s^−1^ in grid cells covering a 339 km^2^ area of the bed and locally as high as 489 m^3^ s^−1^ (Fig. 1b). The basal shear strength was reduced by >5 kPa over a 152 km^2^ area and locally by up to 15 kPa (Fig. 1c). Although this reduction is small compared to the mean value of the gravitational driving stress (73±28 kPa), subglacial sediment weakening induced basal slip by up to 237 m a^−1^, which is 282% faster than the previous day (84 m a^−1^). The corresponding maximum surface velocity was 247 m a^−1^, which is 229% faster than the day before (108 m a^−1^). We also found surface velocity to increase by >5% over an area of 4750 km^2^ (Fig. 1d), whereas basal traction decreased by >5% over an area of only 233 km^2^ (Fig. 1c).

A similar cascading event on 18 July demonstrates the impact caused by lakes draining at higher elevations later in the ablation season. That day, the modelled subglacial drainage network extended more than 100 km inland from the ice margin (Fig. 1e), which is consistent with GPS observations^16^ and observed patterns of acceleration mimicking glacial drainage networks^28^. Basal water fluxes were >80 m^3^ s^−1^ in grid cells covering a 504 km^2^ area of the bed and locally as high as 524 m^3^ s^−1^. The weakening of the bed (Fig. 1f) and the corresponding acceleration of ice flow (Fig. 1g) were similar to those on 21 June, with high-elevation lakes influencing ice flow across a longer drainage path and thus over a larger area, compared to lakes located closer to the margin.

### Membrane stress

We find that basal traction is significantly reduced in regions of the bed where SGL drainage results in basal water fluxes in excess of 80 m^3^ s^−1^ per grid cell in our model (Fig. 2b). This loss of basal traction is compensated by large longitudinal and transverse stress gradients within the ice sheet. The longitudinal gradients in the along (*x*) and across (*y*) ice flow direction can be calculated as $\partial \left( {H\bar R_{{xx}}} \right)/\partial x$ and $\partial \left( {H\bar R_{{yy}}} \right)/\partial y$ where *H* is the ice thickness and $\bar R_{{xx}}$ and $\bar R_{{yy}}$ are the depth-averaged values for the resistive stresses derived from the model’s deviatoric stress tensor (see Methods). The transverse gradients $\partial \left( {H\bar R_{{xy}}} \right)/\partial y$ and $\partial \left( {H\bar R_{{xy}}} \right)/\partial x$ are likewise derived and henceforth we combine and represent them as a single membrane stress. The coordinate system is defined so that the dominant direction of ice flow follows the *x* direction and extension yields positive values of *R*_*xx*_ (see Methods).

The membrane stress is negligible in winter when basal traction alone counters the gravitational driving stress. However, it becomes critically important in summer when it compensates for the sudden loss of basal traction along well-lubricated, high-flux basal drainage pathways (Fig. 2c and Supplementary Fig. 3). This transfer of resistive stress from the basal interface to the ice sheet itself explains why ice flow doubled in speed on 21 June and 18 July (Fig. 1). Crucially, this acceleration occurs not only where the bed is directly affected by lubricating surface water (Fig. 1c, f) but over a much larger area (Fig. 1d, g). This spatially expansive response is a consequence of the non-linear rheology of ice^29^, which concentrates stresses towards the surface where ice is colder and more viscous than ice nearer the bed.

### Tensile shock

**Fig. 3:**
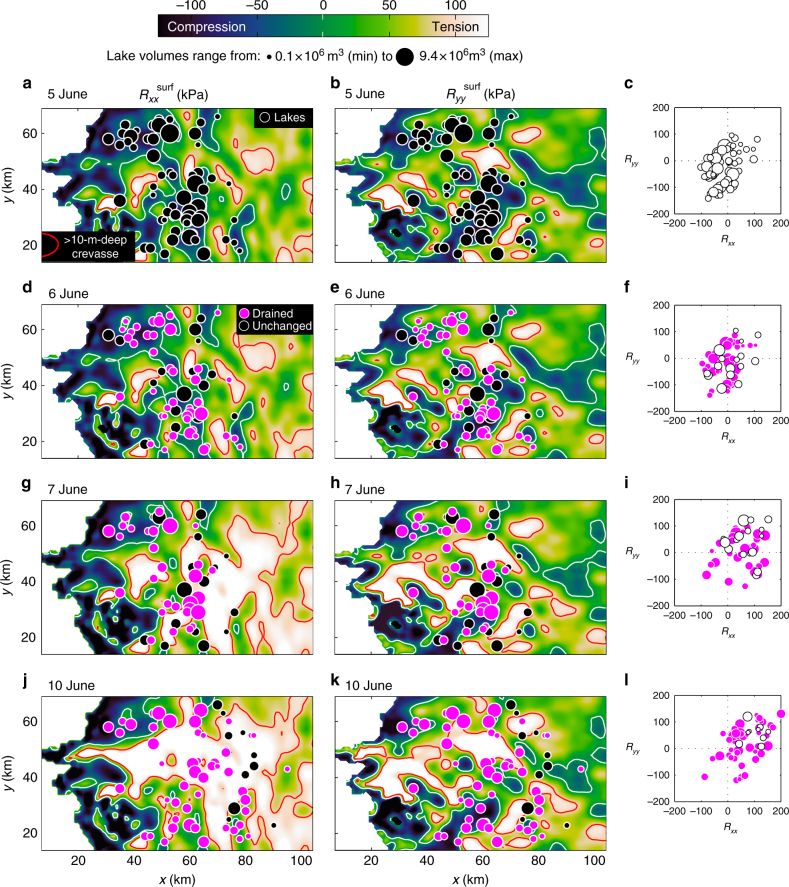
Stress-induced crevasse opening during cascading lake drainage event. **a** Resistive stress in the *x* direction ($R_{{{xx}}}^{\mathrm {surf}}$, kPa) on 5 June. Solid black dots show location and size of 59 supraglacial lakes on the ice sheet that day. White line is the $R_{{{xx}}}^{\mathrm {surf}} = 0$ contour. Red line defines regions where the tensile stresses extend crevasses to a depth of 10 m or more. **b** Same as **a** but showing resistive stress in the *y* direction ($R_{{{yy}}}^{\mathrm {surf}}$, kPa). **c** $R_{{xx}}^{\mathrm {surf}}$ and $R_{{yy}}^{\mathrm {surf}}$ (kPa) at sites where lakes formed. Open black circles are scaled with stored volume of water in each lake. **d** Distribution of $R_{{xx}}^{\mathrm {surf}}$ (kPa) subsequent to the drainage of 43 lakes (magenta dots) on 6 June. Black dots indicate location and size of lakes that had formed but did not drain that day. **e** Same as **d** but showing $R_{{yy}}^{\mathrm{surf}}$ (kPa). **f** $R_{{xx}}^{\mathrm {surf}}$ and $R_{{yy}}^{\mathrm {surf}}$ (kPa) for lakes that drained (magenta dots) and remained intact (open black circles) on 6 June. **g** Distribution of $R_{{xx}}^{\mathrm {surf}}$ (kPa) after 30 additional lakes drained on 7 June (magenta dots). Black dots show 16 lakes that did not drain that day. **h** Same as **g** but showing $R_{{yy}}^{\mathrm {surf}}$ (kPa). **i** Same as **f** but for lakes observed on 7 June. **j** Distribution of $R_{{xx}}^{\mathrm {surf}}$ (kPa) after 51 additional lake drainage events by 10 June. Clouds obscured lake observation on 8–9 June. **k** Same as **j** but showing $R_{{yy}}^{\mathrm {surf}}$ (kPa). **l** Same as **i** but for lakes observed on 10 June. Note how ice flow at lake sites become increasingly extensional as more lakes drain, and how the regions with >10-m-deep crevasses expand as the cascading lake drainage event unfolds

While sudden loss of basal traction induces a membrane stress consisting of stress gradients, the associated increases of the stresses’ absolute values are equally important as they specify where, and to what depth, surface fractures form^30,31^. Hence, we specifically analyse *R*_*xx*_ and *R*_*yy*_ for the 50–150-m-thick top layer of our ice sheet model (henceforth $R_{{{xx}}}^{\mathrm {surf}}{\mathrm {and}}{\kern 1pt} R_{{{yy}}}^{\mathrm {surf}}$), starting with the cascading drainage of 124 lakes on 6–10 June. Before that event, 59 lakes had formed (Fig. 3a, b) with most situated where ice flow was compressional (Fig. 3c), a precondition consistent with previous work^8,10^. On 6 June, however, 43 lakes drained either fully (15) or partially (28), transferring 20 × 10^6^ m^3^ of surface water to the bed. Sixteen lakes remained unchanged (Fig. 3d, e). Due to basal lubrication, ice flow in the *x* direction switched from compressional ($R_{{{xx}}}^{\mathrm {surf}}$ <0) to extensional ($R_{{{xx}}}^{\mathrm {surf}}$ >0) over an area of 186 km^2^ (Fig. 3d). In the *y* direction, a similar switch occurred over an area of 101 km^2^ (Fig. 3e). On 7 June, the next day, two new lakes had formed and 30 out of the 46 lakes observed that day drained either fully (9) or partially (21). The remaining 16 lakes were unchanged (Fig. 3g, h). With an additional 34 × 10^6^ m^3^ of water transferred to the bed, ice flow in the *x* and *y* directions switched from compressional to extensional over areas spanning 786 km^2^ (Fig. 3g) and 571 km^2^ (Fig. 3h) respectively. Due to cloud cover there are no lake observations for 8–9 June. On 10 June, formation of 24 new lakes brought the total number of lakes to 61 and 51 of those experienced drainage (Fig. 3j, k). All but three of these 51 lakes were located in regions where ice flow became extensional in response to basal lubrication (Fig. 3l) and 48 drained completely. A transfer of 40 × 10^6^ m^3^ of water to the bed on 10 June increased extensional ice flow in the *x* and *y* directions by 914 and 606 km^2^ respectively, compared to 5 June, and only 10 lakes remained intact (Fig. 3j, k).

### Crevasse opening and fracture

To examine the mechanical impact, we calculate the depth at which the tensile stress is capable of propagating fractures, i.e. open surface crevasses. As a first approximation, we specify fractures to be confined to regions where $R_{{xx}}^{\mathrm {surf}}$ >0, and their tip to be the depth (*d*) at which this tensile stress is countered by compression due the weight of the overlying ice, yielding $d = R_{{xx}}^{\mathrm {surf}}/\rho g$^31^. We find that tensile stresses do not only propagate existing fractures in areas where ice flow was extensional to begin with; they also initiate new fractures across an expansive area where ice flow was originally compressional (Fig. 3). For example, on 5 June, we find that extensional flow in the *x* direction propagates >10-m-deep surface crevasses parallel to the *y* direction in small, isolated regions covering 18% of the surface of our model (Fig. 3a). Similarly, we find that extensional flow in the *y* direction propagates >10-m-deep surface crevasses parallel to the *x* direction over an area covering 9% of our model. As expected, most lakes are located outside of these crevassed areas. However, as the cascading event unfolds, extensional flow in the *x* direction leads to the propagation of >10-m-deep surface crevasses (transverse to ice flow) in areas covering 20% (6 June), 37% (7 June) and 42% (8–10 June) of the surface (Fig. 3) while extensional flow in the *y* direction propagates equally deep crevasses (parallel to ice flow) over areas of 10% (6 June), 16% (7 June) and 18% (8–10 June) of the surface (Fig. 3).

### Dynamic triggering of lake drainage

**Fig. 4:**
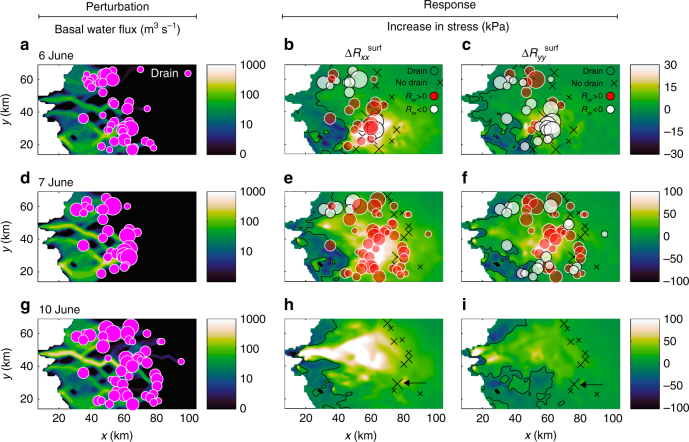
Dynamic triggering of cascading lake drainage. **a** Enhanced flux in the subglacial drainage system caused by 43 lakes observed to drain on 6 June. **b** Change in resistive stress in the *x* direction ($\Delta R_{{xx}}^{\mathrm {surf}}$, kPa) on 6 June relative to the day before. Dots show 30 additional lakes observed to drain the next day (7 June). The colour of dots denotes lakes that drained where ice flow in the *x* direction was extensional ($R_{{xx}}^{\mathrm {surf}}$ > 0, red dots) or compressional ($R_{{xx}}^{\mathrm {surf}}$ < 0, white dots) before the 30 additional lakes drained. The locations of 16 lakes that had formed but did not drain that day are marked with black crosses. **c** Same as **b** but showing change in resistive stress in the *y* direction ($\Delta R_{{yy}}^{\mathrm {surf}}$, kPa). Note how 20 of the 30 lakes drained where ice flow was extensional in either the *x* direction or the *y* direction or in both directions before drainage occurred. The magnitudes of $R_{{xx}}^{\mathrm {surf}}$ and $R_{{yy}}^{\mathrm {surf}}$ are shown in Fig. 3. **d** Basal water flux from the 30 lakes that drained on June 7. **e** $\Delta R_{{xx}}^{\mathrm {surf}}$ associated with the lakes that drained on 7 June. Dots show additional 51 lakes which drained by 10 June (incl. 8–9 June when clouds prevented lake observation). **f** Same as **e** but showing $\Delta R_{{yy}}^{\mathrm {surf}}$. Note how 47 of the 51 lakes drained where ice flow was extensional in at least one direction before drainage occurred. **g** Basal water flux from the 51 lakes that drained during 8–10 June. **h** $\Delta R_{{xx}}^{\mathrm {surf}}$ associated with lakes draining 8–10 June. Crosses mark 10 lakes that remained unaffected by the tensile shock and did not drain. Arrow identifies the only lake to persist from 6 June to 10 June. **i** Same as **h** but showing $\Delta R_{{yy}}^{\mathrm {surf}}$. Note how drainages increasingly occur when ice flow has switched from compressional to extensional and how the location of unchanged lakes on 10 June falls outside the region of the tensile shock

Although our model does not include a lake drainage mechanism, we investigate potential causal mechanisms by comparing the tensile stress perturbation generated by lakes draining in a cluster, e.g. 6 June, with the distribution of lakes observed to subsequently drain in another cluster, e.g. 7 June. For the purpose of this analysis we calculate $\Delta R_{{xx}}^{\mathrm {surf}}\, \mathrm {and}\, \Delta R_{{yy}}^{\mathrm {surf}}$ defined as the change in $R_{{xx}}^{\mathrm {surf}}\, {\mathrm{and}}\, {\kern 1pt} R_{{yy}}^{\mathrm {surf}}$ relative to the day before, as lakes progressively drain. After the 43 lake drainage events on 6 June (Fig. 4a), $\Delta R_{{xx}}^{\mathrm {surf}}$ and $\Delta R_{{yy}}^{\mathrm {surf}}$exceed 25 kPa over areas of 195 and 34 km^2^, respectively. The tensile stress perturbation encircles all but three of the 30 lakes observed to drain on 7 June, i.e. the following day, and 20 out of 30 lake drainages occurred where ice flow had switched from compressional to extensional (Fig. 4b, c). While the model cannot explain why 10 lakes drained in places where ice flow remained compressional despite lakes draining the day before, we hypothesise that these lakes may have drained as a consequence of the tensile stresses generated on the same day by the 20 other extensional lakes. While the temporal (daily) resolution of the lake drainage record allows us to specify changes in ice flow from one day to the next, we cannot specify potential changes occurring in less than a day.

**Fig. 5:**
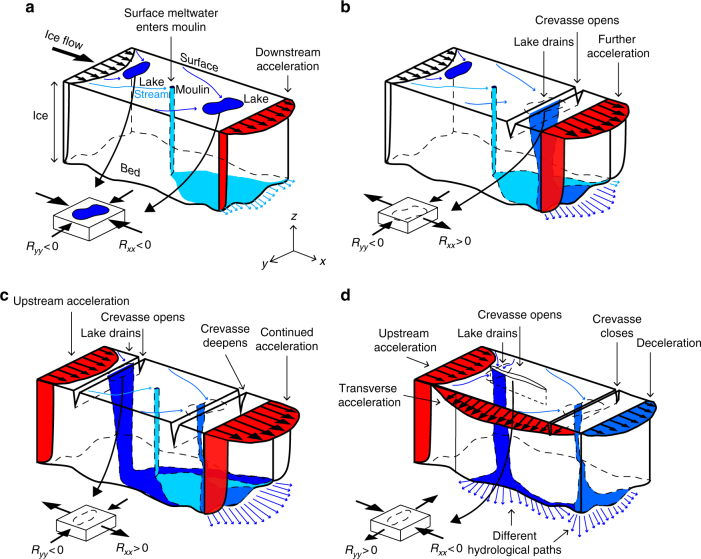
Conceptual model for chain reaction drainage of supraglacial lakes. **a** Schematic illustration showing section of the Greenland Ice Sheet with two lakes situated in compressive ice flow regime (dark blue) and supraglacial melt streams feeding a moulin (light blue). Delivery of meltwater to the bed via the moulin lubricates basal motion, accelerating the downstream ice flow in the *x* direction. **b** A crevasse opens up in the *y* direction because extensional stresses (*R*_*xx*_ > 0) have developed at the surface, triggering a rapid lake drainage event. Additional meltwater injected at the bed accelerates the ice flow further. **c** Lake above the initial perturbation drain because sustained basal lubrication has activated the membrane stress, causing expansion of the extensional ice flow (*R*_*xx*_ > 0) at higher elevations, here illustrated with the opening of a new crevasse in the *y* direction. With tensile stresses growing in both extent and magnitude, a network of draining lakes expands upstream as well as downstream. **d** An alternative scenario in which water is routed in different direction at the bed, causing ice flow acceleration in the *y* direction. In this case, extensional stresses develop in the transverse direction (*R*_*yy*_ > 0), which means that the crevasse beneath the higher lake opens in the *x* direction, i.e. parallel to the main ice-flow direction. The lower crevasse closes because upstream acceleration has caused compression in the *x* direction (*R*_*xx*_ < 0). If acceleration occurs in both directions, crevasses may form transverse as well as parallel to the ice flow direction (not shown)

After the 30 lake drainages on 7 June (Fig. 4d), $\Delta R_{{xx}}^{\mathrm {surf}}$ was >50 kPa over an area of 816 km^2^ and locally as high as 182 kPa (Fig. 4e). Similarly, $\Delta R_{{yy}}^{\mathrm {surf}}$ was almost as high (110 kPa), although less extensive (>50 kPa over 263 km^2^; Fig. 4f). This expansion of the tensile stress perturbation induced extensional ice flow at 48 out of the 51 lakes observed to drain during 8–10 June (Fig. 4e, f). Once those lakes had drained (Fig. 4g), $\Delta R_{{yy}}^{\mathrm {surf}}$ (196 kPa; Fig. 4i) surpassed that of $\Delta R_{{xx}}^{\mathrm {surf}}$ (180 kPa; Fig. 4h), although the latter increased by >50 kPa over a much larger area (925 km^2^ compared to 95 km^2^). The 10 lakes that did not drain during 6–10 June were all located outside the area impacted by this tensile shock (Fig. 4h, i). We find $R_{{xx}}^{\mathrm {surf}}$ >100 kPa over wide regions of our model including the interior (Fig. 3j), whereas $R_{{yy}}^{\mathrm {surf}}$ >100 kPa occurs mainly near the terminus and scattered interior patches (Fig. 3k). The shift from largely compressional ice flow (Fig. 3c) to largely extensional ice flow (Fig. 3f, i, l) explains the formation of extensional fractures beneath lakes that drain. Because these fractures form in a direction which is perpendicular to the applied tensile stress^32^, our model explains the formation of fractures transverse to the dominant ice flow direction when $R_{{xx}}^{\mathrm {surf}} > {\mathrm{0}}$ while fractures parallel to ice flow form when $R_{{yy}}^{\mathrm {surf}} > 0$ (Fig. 5; Supplementary Fig. 4). The latter explains the observed opening of fractures in the ice flow direction^5–7,10^.

## Discussion

Our analysis suggests that the dynamically evolving stress distribution within the Greenland Ice Sheet dictates where SGLs form, and when and why they drain as observed. Due to atmospheric warming, SGLs have become more numerous and larger while expanding to higher elevations^1,12^. Recent studies have suggested that new inland lakes are unlikely to drain rapidly because the surface there lacks pre-existing fractures, asserting that interior meltwater will drain predominantly on the surface and only reach the bed at lower elevations where fractures already provide access to the bed^10,15^. If this access remains stationary as proposed^10,15^, the ice sheet’s response to inland lake expansion would be controlled by the longitudinal coupling length, which specifies the upstream distance over which ice flow accelerates when friction along the bed is locally reduced. While this effect can explain observed variations in ice flow in response to meltwater injected several kilometres downstream^4,33^, the current view is that it is nonetheless insufficient to have a sustained impact on future ice sheet dynamics.

Our model includes the longitudinal coupling effect, but whereas earlier work addressed only the thickness-averaged effect in the ice-flow direction^33,34^, we examine for the first time its variation from one day to another, plus its full impact in 3D (Fig. 2, Supplementary Fig. 3, Methods). The tensile shock induced transiently when lakes drain is significantly larger than the longitudinal coupling effect in previous studies, which did not include lakes or basal hydrology, or the concentration of stresses towards the surface, as reported here. Although the distributed hydrological system in our model is simple (Methods), its interaction with the underlying till layer produces realistic day-to-day variations in ice flow (Fig. 2, Supplementary Fig. 2). The good agreement between model and observation is consistent with a growing body of evidence, which points to distributed and weakly connected hydrological systems linked to till as the dominant control on ice flow rather than channels^35^. With ice flow coupled to a till layer that interacts with a distributed hydrological system, we show how tensile stresses are concentrated towards the surface when water from draining lakes lubricates the bed. We use the term ‘tensile shock’ on the basis of tensile stresses increasing by as much as 182 kPa in a single day. Given that the yield strength of ice is ~100 kPa^32^, this marks a significant and previously unreported perturbation. The magnitude and spatially extensive nature of the tensile shock are underpinned by the observed as well as the modelled variations in ice flow, which are similar (Fig. 2; Supplementary Fig. 2).

**Fig. 6:**
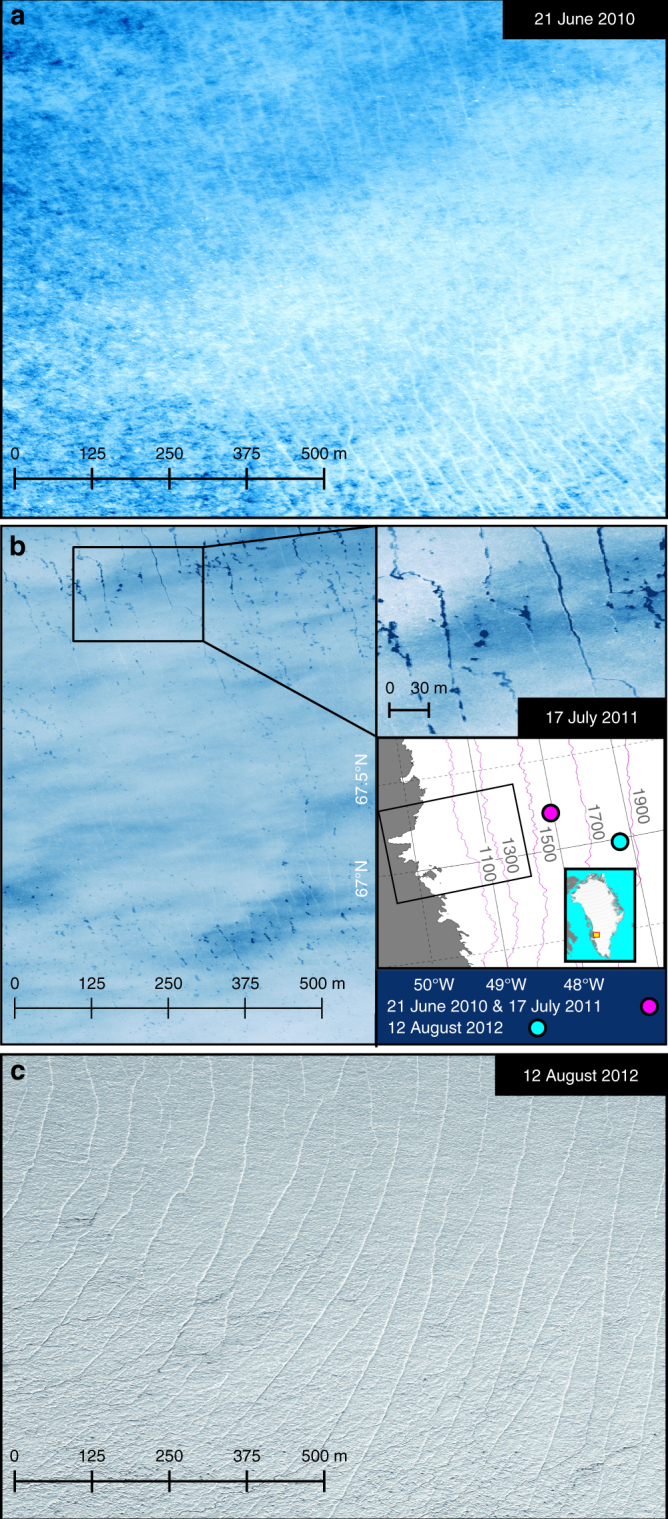
High-elevation crevasses on the Greenland Ice Sheet. **a** Worldview image acquired on 21 June 2010 showing snow-filled crevasses at 1540 m elevation, 95 km inland from the ice sheet margin (48.05°W, 67.20°N) during a cascading lake drainage event (see Fig. 1b–d for contemporaneous model outputs). **b** Worldview image showing water-filled crevasses in the same area on 17 June 2011. Inset shows approximate location of imagery (coloured dots) and domain of numerical model (black box). **c** Worldview image from 12 August 2012 showing high-altitude crevasses at the S10 site (47.165W, 66.985N) at 1800 m elevation, 135 km inland from the margin, which is where Doyle et al.^16^ observed year-on-year increases in ice flow consistent with forcing by supraglacial lakes (Imagery © 2018 DigitalGlobe, Inc.)

Our model outputs suggest initiation of new fractures as well as opening of existing crevasses across much of the ablation area, and over short periods as far as 100 km inland from the margin (Fig. 3). This response is supported by satellite imagery (Fig. 6) showing open surface crevasses 95 km from the ice margin on 21 June, the same day we report a tensile shock from a cascading event (Fig.1). While the interior crevasses observed on 21 June were snow-filled, crevasses observed at the same location a year later were water-filled (Fig. 6b). We also report evidence for water-filled crevasses at 1800 m elevation on 12 August 2012 (Fig. 6c) when crevasses were also observed to open across the Raven Skiway near Dye 2 station at 2100 m elevation^36^. Our model is also supported by satellite laser altimeter data, which shows significant and sudden changes in surface-elevation relief, consistent with opening of crevasses over short periods of time and on a large spatial scale (Supplementary Fig. 5 and Methods). Although crevasses are generally less frequent at the interior than along the margin where ice flow is faster, our model shows >10-m-deep penetration of crevasses in nearly half of the modelled domain when cascading lake drainage events occur (Fig. 3). A previous study showed that lakes as small as a few hundred metres across and a few metres deep may contain sufficient water to drive the propagation of a water-filled crevasse to the base of 1-km-thick ice^37^. Contrary to recent work constrained by mean over-winter strain fields^15^, we find no upper limit on the initiation of fractures in our model when summer meltwater transiently drives large variations in ice flow. Instead, we find that SGLs become widely interconnected through the perturbation they induce on the force balance of the ice sheet when they drain. Of the 663 observed drainage events used to force the model, the vast majority occurred when the bed was well lubricated by water from other draining lakes. Over three quarters of these drainages occurred within seven well-defined events, which explain all major episodes of ice flow acceleration in 2010 (Fig. 2).

Our study is based on a lake drainage record from 2010. While the timing of drainages can vary from 1 year to the next, lakes usually form at the same location year after year^1,14^. In 2010, lakes started to form about 2 weeks earlier than usual, but the cumulative lake volume loss was close to the decadal average at the end of the melt season^1^. Observations do not support the hypothesis that lakes should drain when they reach a critical size or depth^1,6,10^. Lakes often drain in clusters in both space and time^1,14^ and this hitherto unexplained tendency indicates that the cascading events in our model are common. Because we use an observational lake drainage record to force ice flow in our model, we cannot predict lake drainages nor explain the onset of cascading lake drainage events. However, we propose conceptually that cascading events occur as a chain reaction (Fig. 5). The triggering of this chain reaction could be an isolated lake drainage event or the formation of a moulin when a crevasse intercepts a supraglacial meltwater stream. Either way, the delivery of meltwater to the bed may lubricate basal motion locally, as observed near lakes^10^, and ice flow should consequently accelerate along the subglacial hydrological path (Fig. 5a). Lakes situated in compressive basins along this path may drain when the loss of basal traction temporarily induces membrane stresses of sufficient magnitude to initiate and propagate crevasses beneath the lakes (Fig. 5b), causing hydro-fracturing and new surface-to-bed hydrological connections. With more water injected at the bed, basal motion is further enhanced; the ice sheet flows yet faster and more lakes drain as more crevasses open up. To compensate for the increasing loss of friction along the bed, tensile stresses develop laterally as well as longitudinally and upstream as well as downstream (Fig. 5c). At this stage, lakes drain in rapid succession, with crevasses forming transverse (Fig. 5a–c) or parallel (Fig. 5d) to the ice-flow direction, depending on the direction of the tensile stress. The cascading event only abates when the majority of the lakes affected by the tensile shock have drained (Fig. 4).

Cascading lake drainage events are short-lived, lasting only a few days; yet they provide answers to ambiguous and poorly understood attributes of the SGL drainage mechanism. Specifically, they explain why lakes situated in compressive basins often drain via fractures forming by extension^5–7,10^, why lakes often drain in distinct clusters^1,3,38^, why lakes drain through transverse as well as ice-flow-parallel fractures^5–7,10^, and why there is no apparent relationship between the timing of drainage and lake volume or depth^1,6,10^. Moreover, the proposed cascading lake drainage mechanism also explains the precursory activity and uplift recorded by GPS near lakes before they drained^6,10^. The regularity of precursor events at a lake studied since 2006^10^ provides strong observational support for our model, while our model, in turn, demonstrates that precursors not only stem from water supplied to the bed via neighbouring moulins, as proposed^10^, but from the hydro-dynamical inter-connectedness of lakes situated more generally within the same basal drainage path and as far as 80 km apart.

The expansion of SGLs to higher elevations has not only occurred in West Greenland, but across the entire ice sheet at similar rates^12^. The lakes in our studied region are predicted to expand to elevations higher than 2000 m over the next 50 years^19^. Although the size of high-elevation lakes is generally larger than lakes forming at lower elevation^19^, their ability to drain rapidly is a matter of debate. Poinar et al.^15^ argue that the lake drainage mechanism is largely confined to elevations below 1600 m because tensile stresses are insufficient to initiate hydro-fracturing beneath lakes forming at higher elevations. Although lake drainages below this limit in part may explain why Doyle et al.^16^ found ice flow at 1800 m elevation to be consistent with year-on-year increases in lake extent, our study generally does not support the presence of a fixed elevation limit on lakes ability to drain. Instead, we find lake drainages to be confined by the spatial extent of major tensile stress perturbations, which explains why a recent study found lakes above 1600 m elevation to be as likely to drain rapidly as lakes situated at lower elevation^39^. While it is possible that SGLs may form beyond the region affected by tensile shock, it is unlikely that all future SGLs would fall outside this region. A more likely scenario is a tensile shock that progressively expand across larger distances as lakes migrate inland and become more numerous^19^.

While the tensile shock is a transient state, its magnitude and extent explain how crevasses open in regions where ice flow is otherwise compressional and crevasses normally absent or closed. Although this finding is based on numerical modelling, we forced our model with observed SGL volumes and are able to verify the fracturing of ice in our model with contemporaneous imagery showing open surface crevasses at 1500 m elevation and higher. We therefore expect more water to be delivered to previously isolated regions of the interior bed as climate warms^40^ and SGLs expand inland^12^. This delivery will be enhanced by the continued expansion of the ablation area^12,15^ as well as the accumulation area’s decreasing ability to retain meltwater in firn ^41,42^. Water from the surface will, additionally, convert gravitational potential energy as heat at the bed^43^, promoting thawing of frozen and previously isolated regions of the bed, together with easier deformation of warmer basal ice and faster sliding^44^. These ice-flow enhancing processes can only be offset if water from the surface is evacuated in an efficient basal drainage system capable of withdrawing water from its surroundings and thereby increasing the frictional resistance along the bed^45,46^. The latter may, however, be confined to the ice sheet margin. Theoretical work shows that ice velocity remains high farther inland^46^ where thicker ice and flatter surfaces may preclude or limit the development of efficient basal drainage systems^20–22^. This is consistent with observations of efficient basal water systems as far as 30 km inland from the margin^47^ and the decadal slowdown observed up to elevations of about 1000 m^17^. With most lakes forming above that elevation and more lakes forming in larger networks, more surface water is likely delivered to potentially sensitive regions of the ice sheet interior as climate warms.

## Methods

### Ice sheet model

Ice flow was simulated using the CISM, which solves the conservation of mass, thermal energy, and momentum based on the first-order approximation to the Stokes’ equation for ice flow^11,48,49^. The model has a 1 km spatial resolution and was initialised using a standard inversion technique through which surface velocities were iterated towards specified target values. We first prescribed a no-slip basal boundary condition that allowed internal ice deformation to evolve to equilibrium. We then subtracted this value from the target surface velocity so that the model iteratively produced the basal traction and sliding rates needed to fully converge modelled and observed ice flow. The full details of this procedure are given by Price et al.^49^ who used balance velocities as target values. The target values in this work were observed winter 2009/2010 surface velocities derived from TerraSAR-X image pairs^9,23^. With fixed initial model geometry prescribed from a 2008 SPOT surface DEM and a bed DEM produced from ice thickness measured by airborne and ground-based radio-echo sounding data^50^, we converged ice temperature, effective ice viscosity, and ice velocity fields to equilibrium. We obtained an excellent correspondence (*r*^2^ = 0.99, *p* < 0.01) between flow in our initialised model and that observed during the winter 2009/2010 (Supplementary Fig. 1).

### Forcing

To force the model, we used SGL drainage volumes for 2010 produced from semi-automatic mapping of supraglacial water bodies in MODIS imagery. The observations from 2010 (Fig. 2a) were part of a decadal (2002–2012) record. See Fitzpatrick et al.^1^ for the full record and technical details. During the main simulation, which started 15 May 2010 and spanned 6 months, ice thickness, velocity and effective viscosity evolved freely in response to surface water injected at the bed beneath each lake. The injected water volumes were based on MODIS-derived lake losses, with the exception of Lake F, which drained rapidly on 29 June after four days of observed steady lake volume decrease^6^. In this study, we have corrected the lake drainage event on 29 June by incorporating four days of pre-drainage volume losses and reducing the volume that drained on 29 June according to in situ observations. The total water volume from draining lakes was 0.43 km^3^. Water from each lake was injected at the bed in a single grid cell with a time-step lasting 6 h, which made the rate of discharge proportional to the observed reduction in lake volume. We justify this simplifying assumption on the basis that the observational lake drainage record does not inform the duration of discharge^1^. While previous studies have identified both fast and slow modes of lake drainage^7^, the two modes are often defined by different criteria and the reported frequencies thus vary for methodological reasons^39^.

### Evolving basal conditions

To realistically simulate ice flow driven by this forcing we used a dynamic basal sub-model in which sedimentary and hydrological processes interact. This interaction was based on the assumption that the subglacial environment is composed of a layer of till as well as a regional hydrological system capable of transferring large volumes of surface water when SGLs drain. The till layer was described using the Coulomb plastic rheology^51,52^, which is recognised as a novel framework for modelling ice sheets and glaciers flowing over a soft bed^53^ as observed in the Kangerlussuaq sector^24,25^ and elsewhere in Greenland^54,55^. Basal traction in our model therefore evolved according to the quantity of water accommodated by the till layer when SGLs drain. Water was injected at the bed beneath each lake and routed in the hydrological system before interacting with the sediment layer below. The routing of water was based on the D8 steady-state directional algorithm where cells with lower hydraulic potential receive a fraction of the outflow^11^. Although simplified, this approach is consistent with observed tracer velocities of ~1 m s^−1^ in this region^47^, which shows that water is rapidly distributed in the modelled domain and that water from drained lakes may flow as far as 86 km within a day. No assumptions were made with reference to the nature of the basal water system, which simply transfers water according to the slope of the hydraulic potential surface. The sudden input of water from the surface generated high fluxes in the basal water system as well as vertical hydraulic gradients, allowing for an exchange of water between the two systems. Thus, vertical intake of water was most efficient when the horizontal flux in the basal hydrological system was high. The return outflow was specified by hydraulic diffusion controlled by the excess water pressure generated in the till layer when it had accommodated water by expanding pore space. Full details on this dynamic and physically-based basal parameterisation are provided in Bougamont et al.^11^.

### Spring speed-up event

Although 95 lakes drained on 25–28 May (Fig. 2a), their combined volume of 0.03 km^3^ was insufficient to reproduce the observed 'spring speed-up' with the parameterisation used to simulate lake drainage events during the subsequent summer (Fig. 2d). To reproduce the 'spring speed-up' with our model, we injected 0.04 km^3^ of additional water on 24 May (Fig. 2d, ‘red line’). This separate experiment was based on the assumption that water produced from friction and the geothermal heat flux at the bed during the preceding winter (0.04 km^3^) may have been stored subglacially in winter when the thinnest portion of the ice sheet’s margin becomes frozen, causing proglacial discharge to cease^56,57^. Observations from Russell and Leverett glacier show that subglacial meltwater is produced in winter, but not released until spring^56,57^.

### Model validation

When our model was forced with the 2010 supraglacial lake drainage record, ice flow evolved spatially as well as temporally. These intra-seasonal variations, with flow accelerating by up to 400% compared to winter, closely mimic those recorded with GPS at site SHR on Russell Glacier (Fig. 2d). With a three-day running mean applied to smooth uncertainties tied to the timing of lake drainages, we obtain a correlation coefficient of *r*^2^ = 0.83 (*p* < 0.01) from 1 June when lakes alone drive ice flow in our model. When the simulated spring speed-up event in late May is included, the strength of the correlation increases. Furthermore, when modelled surface velocities are averaged over the same 11-day periods used to obtain surface velocities from TerraSAR-X image pairs centred on 19 June, 11 July, 22 July and 11 November, we obtain significant correlation coefficients (*p* < 0.01) of 0.79, 0.92, 0.90 and 0.94, respectively (Supplementary Fig. 2). While the TerraSAR-X tiles do not capture the whole model domain, they cover the portion of the ice sheet where velocities vary the most. The high level of correspondence with observations, spatially as well as temporally, is a unique feature that validates our model. Additional information can be found in Bougamont et al.^11^.

### Ice sheet force balance

The longitudinal gradients in the *x* and *y* directions were calculated as $\partial \left( {H\bar R_{{xx}}} \right)/\partial x$ and $\partial \left( {H\bar R_{{yy}}} \right)/\partial y$ where $\bar R_{{xx}}$ and $\bar R_{{yy}}$ are the depth-averaged values for the resistive stresses *R*_*xx*_ = 2*τ*_*xx*_ − *τ*_*yy*_ and *R*_*yy*_ = 2*τ*_*yy*_ − *τ*_*xx*_ derived from deviatoric stress (*τ*_*ij*_). The latter is defined as the total stress (*σ*_*ij*_) minus the hydrostatic component ((1/3)*σ*_*kk*_*δ*_*ij*_) and thus represents the stress that contributes to ice deformation^40^. The transverse gradients $\partial \left( {H\bar R_{{xy}}} \right)/\partial y$ and $\partial \left( {H\bar R_{{xy}}} \right)/\partial x$ were in a similar manner derived from *R*_*xy*_ = *τ*_*xy*_. The force balance in the *x* and *y* directions are thus^58^:

1 $$\begin{array}{l}\rho gH\frac{{\partial h}}{{\partial x}} = \tau _{{\mathrm{b}x}} - \frac{\partial }{{\partial x}}\left( {H\bar R_{{xx}}} \right) - \frac{\partial }{{\partial y}}\left( {H\bar R_{{xy}}} \right)\\ = \tau _{{\mathrm{b}x}} - {{{\rm {membrane}}}}{\kern 1pt} {{{\rm {stress}}}}_{x}\end{array}$$

2 $$\begin{array}{l}\rho gH\frac{{\partial h}}{{\partial y}} = \tau _{{\mathrm{b}y}} - \frac{\partial }{{\partial y}}\left( {H\bar R_{{yy}}} \right) - \frac{\partial }{{\partial x}}\left( {H\bar R_{{xy}}} \right)\\ = \tau _{{\mathrm{b}y}} - {\mathrm{membrane}}\,{\mathrm{stress}}_{y}\end{array},$$

where *ρ*, *g* and *h* respectively denote ice density, gravitational acceleration and surface elevation. The left-hand side is the driving stress. The first term on the right-hand side is basal drag tied to frictional resistance at the bed, which directly opposes the driving stress. The second and third terms on the right-hand side are longitudinal and transverse stress gradients, which either oppose or act in cooperation with the driving stress. These gradient stresses make up the membrane stress.

### Crevasses

Open crevasses were detected in satellite imagery (Fig. 6) and the timing of opening was examined using GLAS05 ICESat-1 elevation data acquired in repeat orbital tracks. We used data from spring and summer 2005 and 2006 (Supplementary Fig. 5), with no other year in the 2003–2009 ICESat data record providing repeat seasonal overpasses in the studied region. The elevation data were spatially subset and filtered to remove weak or invalid returns. Within-footprint elevation relief (ER) was determined from the root mean square best Gaussian fit of the return waveform, with the Full Width Half Max (FWHM) of the Gaussian fit converted from nanoseconds to metres after accounting for the length of the outgoing pulse^59^:

3 $$\begin{array}{l}{\mathrm {ER}} = \left( {{\mathrm {FWHM}}_{{\mathrm {return}}} - {\mathrm {FWHM}}_{{\mathrm {transmit}}}} \right)\\ \times \left( {0.15{\kern 1pt} {\mathrm {m}}/{\mathrm {ns}}} \right)\end{array}$$

Terrain complexity was examined using Gaussian decomposition to identify the number of modal peaks within the return waveforms^59^.

Overpasses on 28 February and 22 March 2005 show an ice sheet surface that was flat and gently sloping, with near zero ER (Supplementary Fig. 5a). A repeat pass-over on 22 May revealed little change in ER compared to 22 March, but on 29 May and 1 June the ER had increased to ~3 m along two tracks that followed the 1700 m elevation contour, 110 km inland from the ice sheet margin near 67.5°N and 47.5°W (Supplementary Fig. 5b). On 16 June, the ER had intensified to >10 m at 1400 m elevation north of 67.5°N and slightly less (~3 m) at lower elevations near 67°N. The observed increase in ER was accompanied by changes from planar to complex surfaces, often within days (Supplementary Fig. 5b). Although ER may increase from the exposure of rough icy surfaces in places where snow disappears, the observed changes in ER cannot be exclusively attributed to snow melt because they occur at low (<1000 m) and high (>1700 m) elevations simultaneously and at different locations from one year to the next. The increase in ER and the accompanying abrupt change from planar to complex surfaces is consistent with crevasses becoming deeper and more densely spaced locally. Significant elevation differences between overlapping overpasses separated by only a few days highlight the short temporal scale of the dynamic crevasse-opening mechanism (Fig. 5, Supplementary Fig. 4). The smaller ER observed south of 67°N in late May and early June is in good agreement with observations showing that lakes did not form in that region until 5 June that year^1^. In 2006, we observed overall similar evolution of ER, although significant short-term elevation change also occurred south of 67°N, where crevasse may have opened in response to lakes starting to form on 11 May^1^ (Supplementary Fig. 5c, d). The onset, duration and total number of lakes observed in 2006 were similar to those of 2010^1^.

### Data availability

The model code used in this study is based off the Community Ice Sheet Model, which is freely available (https://github.com/CISM). All input datasets and model development code are available on request from the corresponding author.

## Electronic supplementary material

Supplementary Information
