# Supplementary material for: Cascading lake drainage on the Greenland Ice Sheet triggered by tensile shock and fracture
Source: Nat Commun. 2018 Mar 14;9:1064. doi: 10.1038/s41467-018-03420-8 (PMC5852115; doi:10.1038/s41467-018-03420-8)
Supplement: Supplementary file 1 — Supplementary Information [file 41467_2018_3420_MOESM1_ESM.pdf]

Cascading lake drainage on the Greenland Ice Sheet triggered by tensile shock and fracture

Christoffersen *et al.*

Supplementary Information

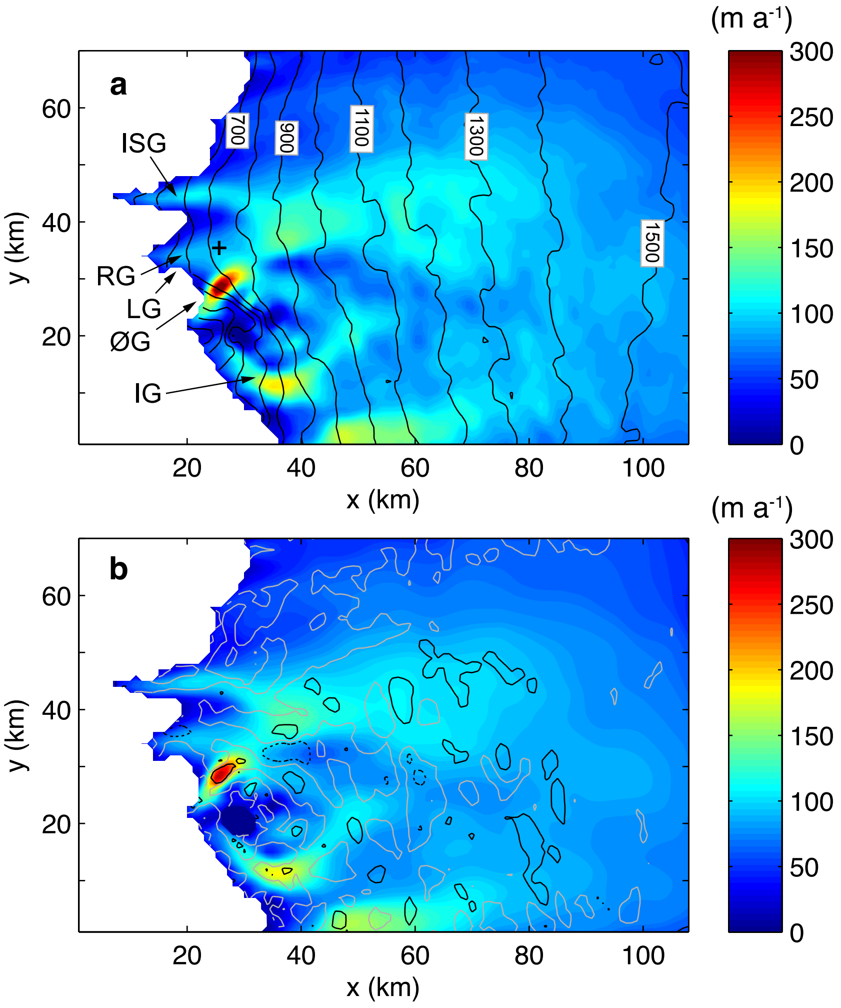

Supplementary Figure 1 I Model initialisation. **(a)** Winter 2009-10 observed surface velocities for the Kangerlussuaq sector of the Greenland Ice Sheet (m a^{-1}, colour scale) from MeSUREs (*ref. 23*). Black contours show surface elevation (m) above sea level. Domain includes Isunnguata Sermia (ISG), Russell Glacier (RG), Leverett Glacier (LG), Ørkendalen Glacier (ØG), Isorlersuup Glacier (IG) and site SHR (+) on the K-transect. **(b)** Initial surface velocities in numerical model (m a^{-1}, colour scale) with contours showing difference between model and observation: +10 m a^{-1} difference (black), 0 difference (light grey) and -10 m a^{-1} difference (dashed black).

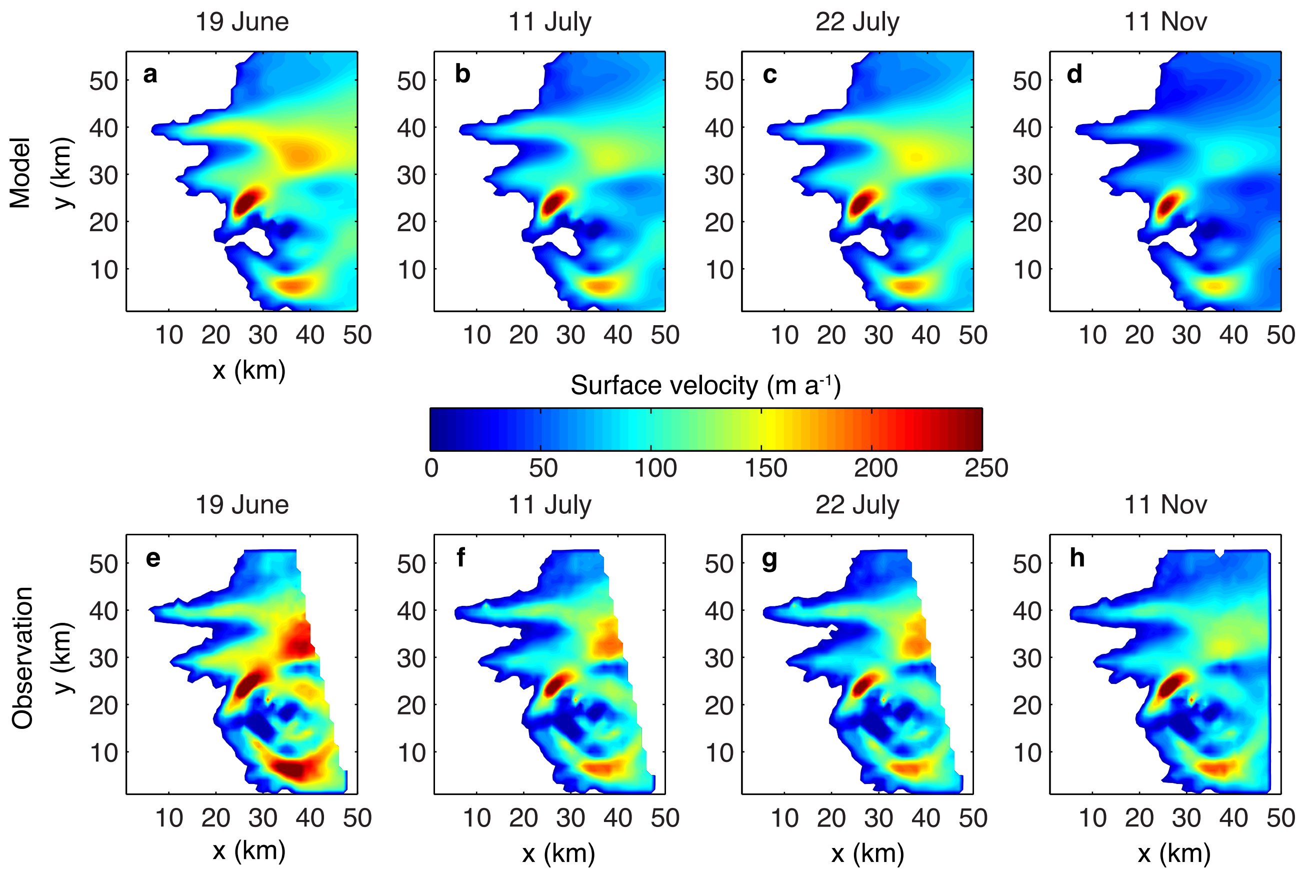

Supplementary Figure 2 I Model validation. (a-d) Modelled surface velocity averaged over 11-day time intervals centred on 19 June, 11 July, 22 July and 11 November (a-d). **(e-h)** observed surface velocity from TerraSar-X satellite data acquired over the same periods. Good correspondence between model outputs and observations yields r^2=0.79 ($p<0.01$) on 19 June, r^2=0.92 ($p<0.01$) on 11 July, r^2=0.90 ($p<0.01$) on 22 July and r^2=0.94 ($p<0.01$) on 11 November. Modified from *Bougamont et al.* (ref. 11).

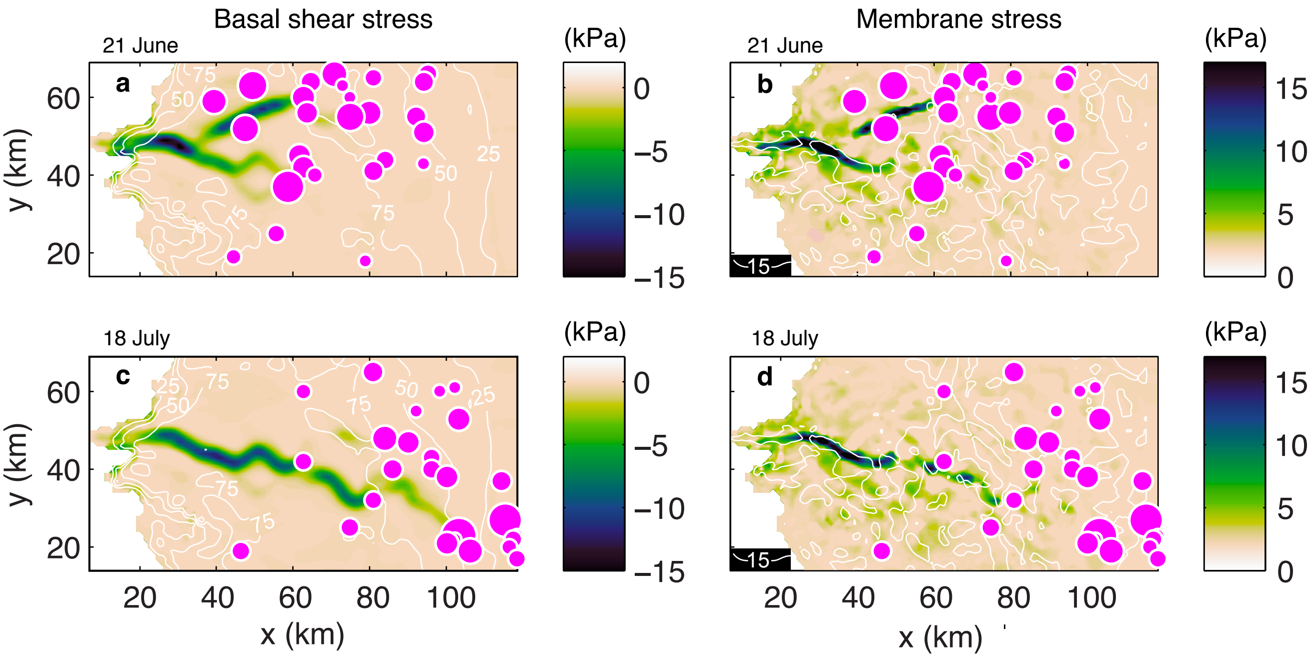

Supplementary Figure 3 l Basal traction and activation of the membrane stress. (a) Change in basal shear stress (kPa) one day after drainage of 26 lakes (solid dots) on 21 June. White contours show absolute values of shear stress prior to drainage. **(b)** Same as (a) but showing corresponding change in membrane stress. **(c)** Change in basal shear stress (kPa) one day after drainage of 26 lakes (solid dots) on 18 July. White contours show absolute values of shear stress prior to drainage. **(d)** Same as (c) but showing corresponding change in membrane stress.

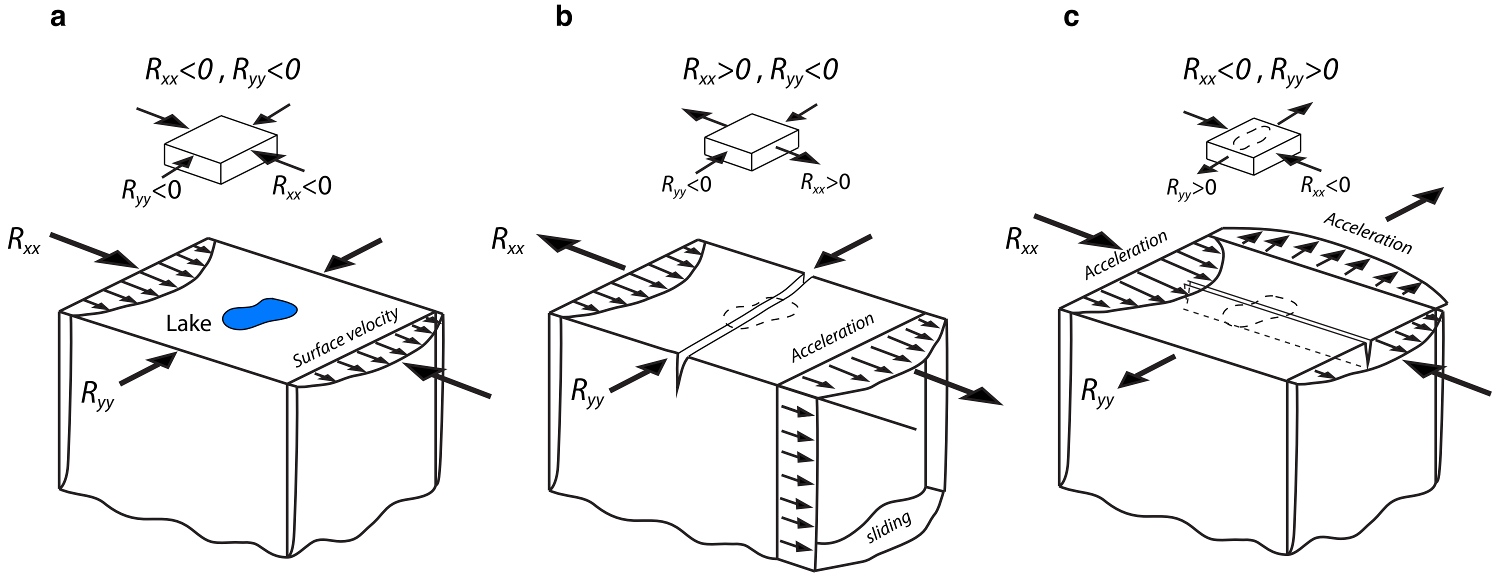

Supplementary Figure 4 l Dynamic triggering of supraglacial lake drainage via different types of fractures. (a) Supraglacial lake (blue) in a region with a compressional flow regime (R_{xx}>0, R_{YY}>0) **(b)** Acceleration of ice flow due to enhanced basal sliding induced by lubrication from lake draining farther upstream. If the perturbation produces extensional flow in the x-direction (R_{xx}>0), the lake is likely to drain via a fracture forming transverse to ice flow. **(c)** If the perturbation produces extensional flow in the y-direction (R_{yy}>0), the lake is likely to drain via a fracture forming parallel to ice flow.

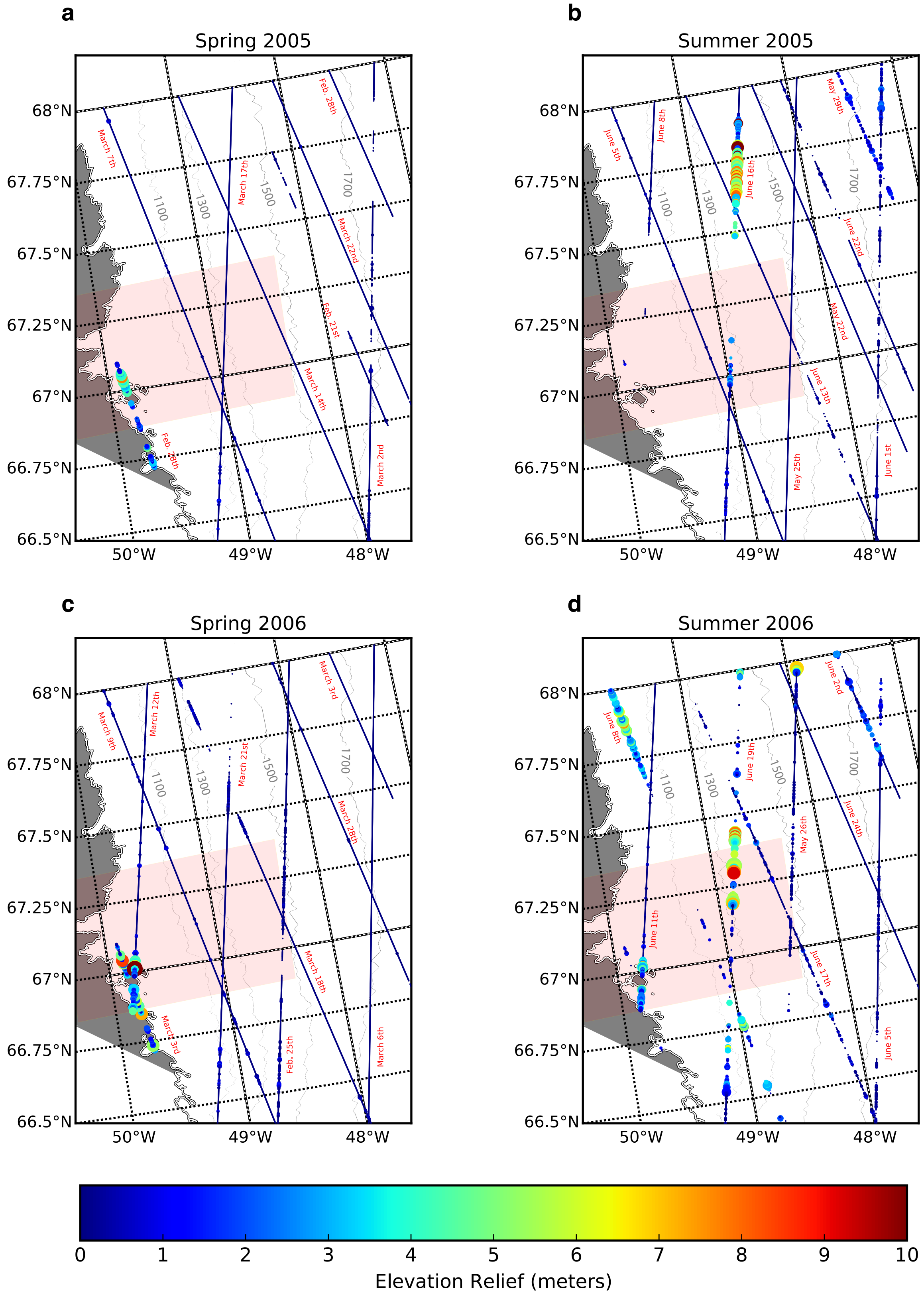

Supplementary Figure 5 l Changes in surface relief when crevasses form. ICESat-1 GLAS laser altimeter measurements (coloured dots) showing within-footprint elevation relief (colour scale) from repeat satellite overpasses in 2005 (top) and 2006 (bottom). The size of each dot is scaled to the observed complexity of the terrain, with small dots indicating a planar surface and larger dots a more complex terrain. Dates denote the time of each satellite pass-over and grey lines are the 1100 m, 1300 m, 1500 m, and 1700 m elevation contours. Measurements from overpasses in February and March (**a** , **c**) show a gently sloping ice sheet surface and a within-footprint elevation relief close to zero. Measurements from repeat overpasses in May and June (**b, d**) show significant increases in within-footprint elevation relief as well as terrain complexity. The changes are interpreted to reflect formation of crevasses. The significant differences between overlapping overpasses separated by only a few days, e.g. between 13 June and 16 June 2005, highlight the short temporal scale of the dynamic crevasse opening mechanism. See Methods for details.
